# 3D Tdoa Problem Solution with Four Receiving Nodes

**DOI:** 10.3390/s19132892

**Published:** 2019-06-29

**Authors:** Javier Díez-González, Rubén Álvarez, Lidia Sánchez-González, Laura Fernández-Robles, Hilde Pérez, Manuel Castejón-Limas

**Affiliations:** 1 Department of Mechanical, IT and Aerospace Engineering, Universidad de León, 24071 León, Spain; 2 Positioning Department, Drotium, Universidad de León, 24071 León, Spain

**Keywords:** TDOA, sensor networks, hyperboloids, node distribution, genetic algorithms

## Abstract

Time difference of arrival (TDOA) positioning methods have experienced growing importance over the last few years due to their multiple applications in local positioning systems (LPSs). While five sensors are needed to determine an unequivocal three-dimensional position, systems with four nodes present two different solutions that cannot be discarded according to mathematical standards. In this paper, a new methodology to solve the 3D TDOA problems in a sensor network with four beacons is proposed. A confidence interval, which is defined in this paper as a sphere, is defined to use positioning algorithms with four different nodes. It is proven that the separation between solutions in the four-beacon TDOA problem allows the transformation of the problem into an analogous one in which more receivers are implied due to the geometric properties of the intersection of hyperboloids. The achievement of the distance between solutions needs the application of genetic algorithms in order to find an optimized sensor distribution. Results show that positioning algorithms can be used 96.7% of the time with total security in cases where vehicles travel at less than 25 m/s.

## 1. Introduction

Positioning is an essential factor for the correct navigation and location of vehicles. Accuracy in calculating positions has commonly determined the fields where positioning has been applied. High technological levels have been achieved when uncertainty has been sufficiently reduced. The usage of localization methods has evolved throughout the last few years from a reference object to precision applications such as farming, indoor navigation or manufacturing environments.

Positioning systems can be divided into those based on time measurements and those that measure different properties, such as angle of arrival (AOA) [1,2] or received signal strength indicators (RSSIs) [3,4,5]. Among them, time measurement systems are the most used, due to availability, accuracy, simplicity and robustness. In this category, TOA (time of arrival) systems [6,7] such as GPS, GLONASS or Galileo, and TDOA (time difference of arrival) systems [8,9] such as LORAN, OMEGA or WAM (wide area multilateration) [10]—which is highly widespread in aircraft environments—are considered.

TOA systems measure the total time-of-flight of a signal between a transmitter and a receptor. They require time synchronization between the transmitter and receptor and their accuracy is highly dependent on clock drift in this synchronization. These time-of-flight measurements lead to equations of three-dimensional spheres centered on the transmitter, representing possible locations of the vehicle in the space.

In contrast, TDOA systems measure the relative times between signal arrival for two different receivers. In this case, synchronization is optional, differentiating asynchronous (A-TDOA) [11] and synchronous (S-TDOA) [12] systems, which can lead to a reduction in error levels. In such a scenario, time difference measurements generate the equations of hyperboloids, whose intersection determine the position of the vehicle.

A number of *n* equations can be obtained from *n* different receivers in TOA systems due to global time measurements in each receptor. In contrast, relative measurements in TDOA systems must consider different combinations for the time difference of arrival measurements that originate from each pair of receivers. These combinations do not allow repetitions in pairs 1-2 or 2-1, mainly due to the duplicity of results. However, it has been proven that from a set of *n* different receiving sensors in a TDOA problem, only a number of (*n*-1) independent equations can be obtained. In addition, the biggest limitation in the equations of spheres and hyperboloids is that they are considered to be non-linear equations. This produces a non-direct resolution of the positioning problem through these equations, a fact that causes the intersection of spheres or hyperboloids to not have a unique solution in the space. Two different solutions can therefore be obtained that cannot be distinguished through mathematical criteria.

According to rigidity theories on positioning systems [13], to completely determine the unequivocal location of an object in a three-dimensional space a minimum of four receptors are necessary in TOA systems, with a minimum of five in cases of TDOA systems. This disposition would guarantee one single solution for the positioning problem. However, global positioning systems such as GPS do not necessarily require an additional satellite for the calculation of the position, since the distances between emitter and receptor are so far-off that the sphere equations generated allow the incorrect solution to be discarded as incoherent or too separate from the previous position of the vehicle.

This problem, apparently solved in global navigation systems, poses a great importance in local positioning systems (LPSs) [14,15] such as those used in precision applications (e.g., indoor navigation or aircraft landings in nowadays airports). This is due to the proximity between the two different solutions in these cases, so that any solution can be discarded with a stable generalized criterion. In this article, a new criterion is proposed to solve this geometric problem based on the properties of certain positioning algorithms. TDOA algorithms will be considered due to their great usage in LPSs [16].

In Section 2, the TDOA positioning problem is described. In Section 3, some different algorithms are presented to solve the TDOA problem in real-time, while in Section 4 fictitious point studies based on TDOA algorithms are developed to guarantee a four-receiver TDOA solution, and the convergence sphere is defined. We show that computers have great difficulty processing convergence spheres in Section 5, and a new parameter to process the convergence radius is proposed in Section 6. Section 7 develops an optimized node localization to solve the 3D TDOA problem. The article concludes with a presentation and analysis of the results obtained and by extracting conclusions from the completed work.

## 2. The TDOA Problem

TDOA systems are based on difference time measurements between the signal arrival to different nodes or sensors in a network. These measurements can be converted to difference of distances by multiplying these times by speed emission of the radioelectric waves (*c*). 

This leads in *Euclidean Geometry* to the next equation:

(1) $$R_{ij}=d_{ij}=d_{Ii}-d_{Ij}\;=\sqrt{{\left(x_{I}-x_{i}\right)}^{2}+{\left(y_{I}-y_{i}\right)}^{2}+{\left(z_{I}-z_{i}\right)}^{2}}\;-\sqrt{{\left(x_{I}-x_{j}\right)}^{2}+{\left(y_{I}-y_{j}\right)}^{2}+{\left(z_{I}-z_{j}\right)}^{2}}+h\left(0,\sigma \right)=ct_{ij}+h\left(0,\sigma \right)\;$$

where $d_{Ij}$ is the distance difference between receivers *i* and *j*—which is the result of multiplying the actual time difference of arrival ($t_{ij}$) and adding a white noise, h(0,σ), that considers atmospheric instabilities and time error measurements. This noise is related to signal transmission and measurement of times, which cannot be controlled by TDOA algorithms and so is not considered in this paper. In addition, $\left(x_{I},\;y_{I},\;z_{I}\right)$ are space coordinates of the vehicle that are being positioned and $\left(x_{i},\;y_{i},\;z_{i}\right),\;\left(x_{j},\;y_{j},\;z_{j}\right)$ are coordinates of the nodes *i* and *j*, respectively, which receive the positioning signal. These equations correspond with hyperboloids that cannot be solved in an analytic direct process. Thus, numerical methods must be used to determine the problem.

## 3. Algorithms for TDOA Problem Resolution

Non-linear equations of hyperboloids must be treated in order to address the TDOA problem resolution. Generally, two main methodologies have been considered: those based on hyperboloids intersection properties with closed-form solutions, and those based on numerical methods, which offer a progressive reduction on the error gradient derivation in successive approximations leading to the final solution. Although these methods could be considered analogous, they use different properties and methodologies. However, both of them share the qualification that a univocal TDOA problem resolution must use at least five different sensors. Hence, from now on, a combined study with a method for each case is proposed to solve the TDOA problem with only four beacons.

Bucher and Misra [17] proposed a method based on the properties of the intersection of hyperboloids. They show that hyperboloid intersections can always be contained in a plane. This process increases the freedom to the problem by one degree, since a number of *n* receivers generate a number of (*n*-1) independent hyperboloid equations and (*n*-2) independent intersection planes are obtained using this methodology. That means that to solve the 3D TDOA problem linearly, where three planes are needed, we still have to use five different receivers. Nevertheless, the fact that the intersection of two different hyperboloids is contained in a plane makes the process of obtaining this plane equation independent from the original hyperboloid equations. As a consequence, the intersection of two planes (four nodes) resulting in a line of possible vehicle localizations can be verified in any hyperboloid to finally get the two solutions that are achieved in TDOA problems with four beacons (*i*, *j*, *k*, *l*). This methodology leads to two different solutions that for LPS cannot be discarded by any assumable criterion.

The other method would be based on applying a Taylor approximation truncated on first order to linearize the equations and allow a real-time solution to the problem. In this way, a point with enough proximity to the final solution $\left(x_{0},\;y_{0},\;z_{0}\right)$ from which a process of sequential iterations will be started is selected. These steps will finally allow the vehicle localization to be obtained through a matrix where the range differences are considered as follows:

(2) $$R_{ij}=ct_{ij}=R_{ij_{0}}+\frac{\partial R_{ij}}{\partial x}\Delta x+\frac{\partial R_{ij}}{\partial y}\Delta y+\frac{\partial R_{ij}}{\partial z}\Delta z\;$$

where $R_{ij}$ is the value of the distance difference in the approximation point, and $\frac{\partial R_{ij}}{\partial x}$, $\frac{\partial R_{ij}}{\partial y}$ and $\frac{\partial R_{ij}}{\partial z}$ are partial derivatives of the range differences, particularized for the values of the approximation point. Applying this very same process to the other two nodes k and l with reference to the node *i,* $R_{ik}$ and $R_{il}$ can be estimated. This leads to the following matrix system:

(3) $$\Delta R=\left(\begin{array}{ccc} \begin{array}{c} \frac{\partial R_{ij}}{\partial x}\\ \frac{\partial R_{il}}{\partial x}\\ \frac{\partial R_{ik}}{\partial x} \end{array} & \begin{array}{c} \frac{\partial R_{ij}}{\partial y}\\ \frac{\partial R_{il}}{\partial y}\\ \frac{\partial R_{ik}}{\partial y} \end{array} & \begin{array}{c} \frac{\partial R_{ij}}{\partial z}\\ \frac{\partial R_{il}}{\partial z}\\ \frac{\partial R_{ik}}{\partial z} \end{array} \end{array}\right)\left(\begin{array}{c} \Delta x\\ \Delta y\\ \Delta z \end{array}\right)\;$$

where ∆*R* is the range differences matrix, *H* is the partial derivative matrix (commonly known as visibility matrix) and *P* is the position variance matrix. Therefore, we can express the matrix system as follows:

(4) HΔP=ΔR 

This equation is usually solved through the least squares method [18], as described below:

(5) $$\Delta P={\left(H^{t}H\right)}^{-1}H^{t}\Delta R=\left(\begin{array}{c} \Delta x\\ \Delta y\\ \Delta z \end{array}\right)\;$$

The coordinates of the solution point in the first iteration would be the result of adding all the approximation coordinates to the increments obtained. After several iterations, the residual error is reduced, reaching convergence with the real solution once it has become lower than the desired precision. However, the convergence of this method depends on the initial position chosen as the start of the first iteration [19]. Regarding the resolution of the TDOA problem, four receiving sensors do not always guarantee the convergence of the method and, if produced, this can affect any of the two possible solutions (which prevents us from knowing whether the position calculation is correct). However, in contrast with the former method, the calculation of the position now guarantees a single solution instead of two possible answers.

## 4. Fictitious Point Method

Of all the methods proposed so far, it is not possible to conclude whether the TDOA System can be applied to LPS systems with four nodes with enough confidence to guarantee the correct calculation of the position. Nevertheless, it is possible to affirm that successive approximation methods do guarantee convergence—if produced—towards one of the possible of the solutions.

**Figure 1:**
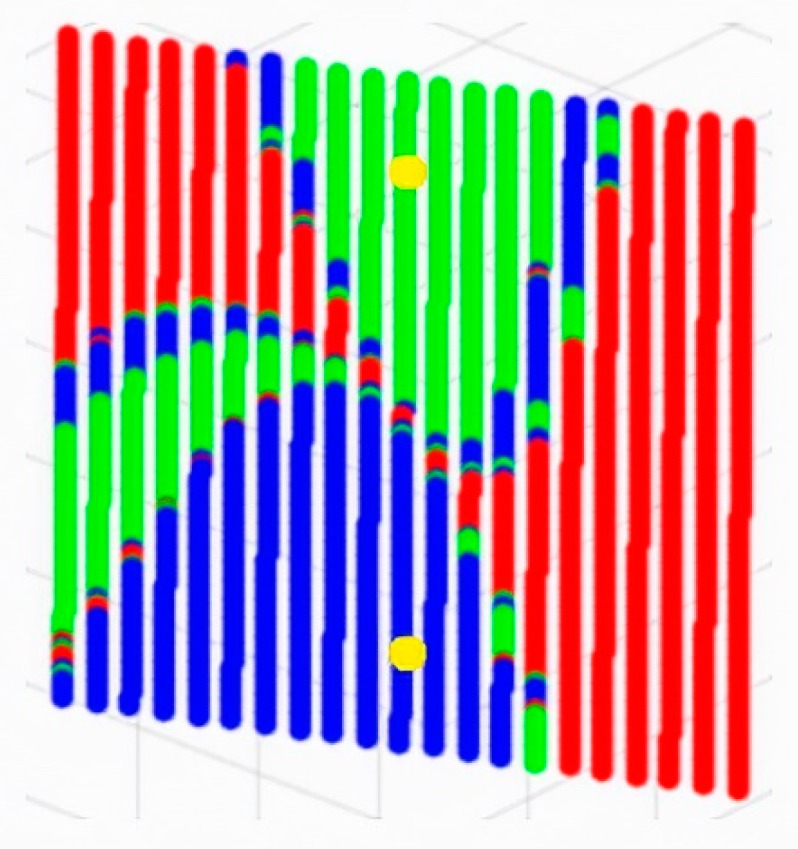
Plane of convergence containing the two solutions of a four-beacon TDOA problem.

This means that if there were any way to ensure that the convergence occur toward the correct solution, the method would allow the problem with to be solved with four sensors. In a scenario where the process is convergent and highly dependent upon the initial point of the iterations, it is safe to say that when this initial point is close enough to the solution (i.e., the previous solution of the vehicle), the convergence should always take place toward the correct solution. To prove this statement, the behavior of any point located at a plane containing the two possible solutions is going to be proven for the TDOA problem. The solution has been calculated by applying the successive approximation method to these initial points, as presented in Figure 1.

Figure 1 represents the two potential solutions (in yellow) to the positioning problem. Their surroundings are color-coded (blue and green) in accordance with which solution these neighbors converge at. Regions in red show an absence of convergence with the successive approximation method. As it is a 3D positioning system, it is necessary to extrapolate the same reliable zone (for calculating the position with four nodes) as a 3D space to find the solution to the problem. The resulting figure would necessarily be a sphere, since the vehicle can move in any direction, with the solution as the center.

**Figure 2:**
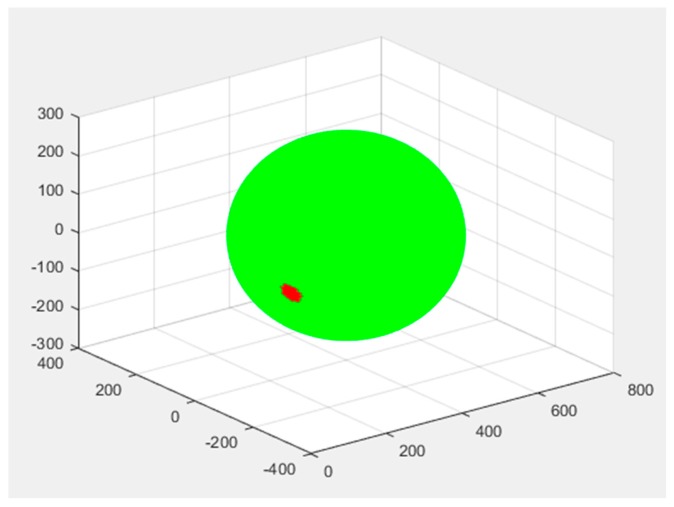
Critical convergence sphere: Surface points in green are good initial position estimates that make the successive approximation method converge at the solution in the center. Points in red fail to make the approximation method converge. This figure displays the first appearance of such instabilities when increasing the radius of the sphere from zero to a critical value marked by the appearance of these defective seeds. The axes represent the 3D environment around the solutions and their units are adimensional due to the illustrative purpose of the figure.

Figure 2 displays the first instabilities appearing in the surface of the convergence sphere. This guarantees that, at a maximum radius, all points on the surface of the sphere are convergent towards the inner (correct) solution. 

In this scenario, a configuration with four receiving sensors within the coverage can be defined as reliable if the distance from the initial point to the solution is inferior to the minimum radius of convergence for all points of that volume.

## 5. Convergence Parameter Modification

The convergence radius is calculated from an evaluation of the points from the sphere centered on the desired solution. In the case that all these points converge towards the inner solution, the value of the radius of convergence increases until there exists divergence at any point. This gradual process of incrementing the radius involves a higher number of calculation points for each iteration process, which cannot be assumed in a reasonable time.

Taking this into account, a different way to determine convergence is proposed in this paper. The surroundings of the solutions find a region in which convergence is not reached in the fictitious point method. This region is considered to be the border between the two intervals of convergence when sequential approximations are used to find the solution. Thus, if the two solutions could be separated enough, the discontinuity region could be ousted from the solutions, which would increase the convergence radius. 

This problem leads to an association between the convergence radius and the distance between solutions. To show this, the convergence radius and distance between solutions are calculated in a representative number of points for the coverage area of a concrete node distribution. For this purpose, the spatial volume where positioning is going to be used to locate a target is divided into small steps in the three Cartesian coordinates, in order to evaluate the convergence radius and the distance between solutions at each point and show the correlation between the parameters.

The correlation between these two factors is shown in Table 1 and reaches a value of 0.999. This value allows us to conclude that any variation of these two parameters will be strongly related to the other.

In this sense, the new parameter can be calculated, leading us to a new conclusion: The maximization of the distances between solutions for every coverage point of a concrete node distribution leads to an increase of the interval of confidence of the sequential approximation method to solve the four-beacon TDOA problem.

However, for a determined sensor distribution, the distances between solutions in the four-beacon TDOA problem are fixed. Hence, in order to maximize this parameter, a search for the optimum node distribution is needed. This will lead to maximizing the convergence interval of the algorithm.

## 6. Optimization of the Node Distribution for the Four-Beacon TDOA Problem

The calculation of the distance between solutions allows us to process the radius of convergence in a reasonable period of time. Due to the geometric properties of the intersection of hyperboloids, some particularities should be considered when a maximization of this parameter is performed. 

**Figure 3:**
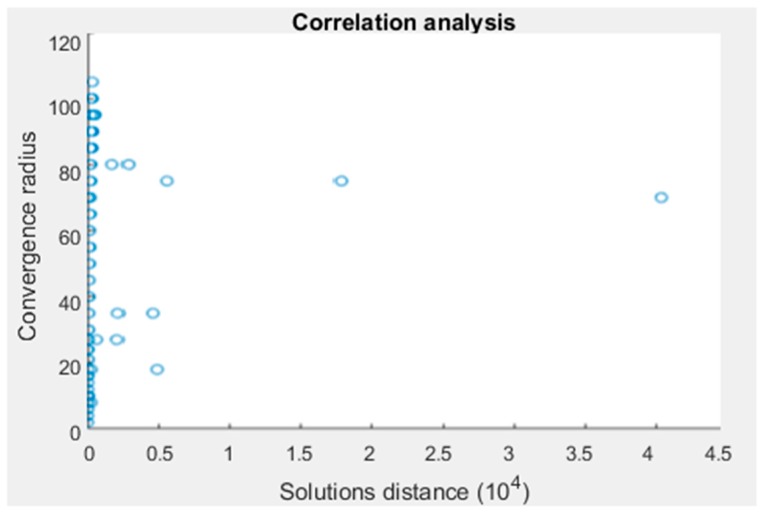
Outliers of the correlation between radius of convergence and distance between solutions.

A set of points with high distance between solutions values is shown in Figure 3. These points do not have a direct correlation with the radius of convergence, but they represent less than 5% of the total points. This is due to a near-tangent condition in the intersection of two different branches of the hyperboloids. The effect of this condition is the separation of the two solutions.

A different circumstance occurs when it is observed at a distance between solutions of 0—which is the tangent case—and does not permit a correlation with the convergence radius in this context. The separation of the solutions or the existence of only one of them modifies the convergence problem in a four-beacon TDOA problem. The problem is converted into a different case of convergence where more receivers are concerned.

However, these points imply a great distortion for the comparison of statistical properties of the node distributions based on the distance between solutions in the four-beacon TDOA problem. In order to remove this type of point, a filter is applied before performing an optimization. The filtering process is run in two different steps:

(1) elimination of points where the distance between solutions is equal to 0;
(2) introduction of a parameter to remove the outliers where the distance between solutions is aberrant, without losing the representativeness of the sample values.

This second step is controlled with the parameter *r*, which measures the correlation between the mean of the sample values of the distance between solutions and both of their ends as a dispersion indicator.

(6) $$r=\frac{\max\left(dist_{sol}\right)-\min\left(dist_{sol}\right)}{mean\left(dist_{sol}\right)}\;$$

**Figure 4:**
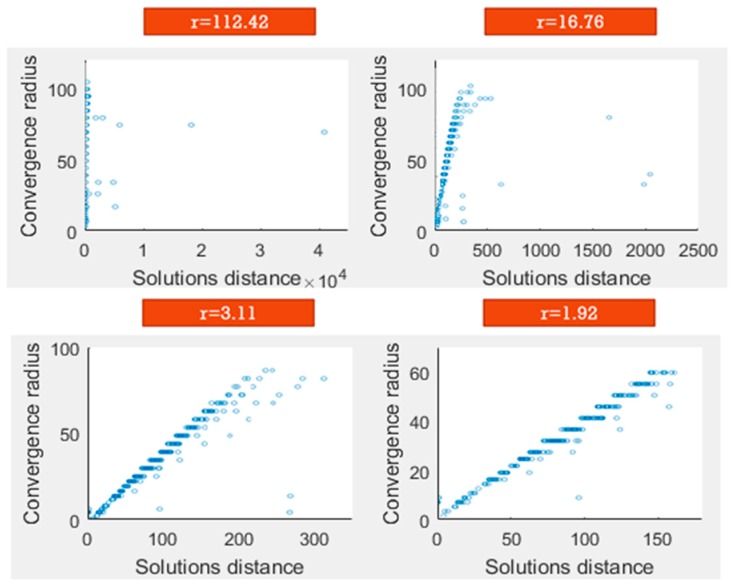
Sequential reduction of the r-correlation factor. The outliers are removed with this iteration process. The remaining distribution (r = 1.92) does not present outliers.

It is concluded that node distributions with outliers show values of *r* above 2.5, so that an elimination of points, such as the filtering process in Figure 4, must be performed until a value of *r* smaller than 2.5 is obtained. In this case, the methodology followed is based on standard deviation. In the first steps of the filter, the standard deviation has high values as a consequence of the outliers. This circumstance allows us to define the limit of the points discarded as a sum of the media and the standard deviation. 

**Figure 5:**
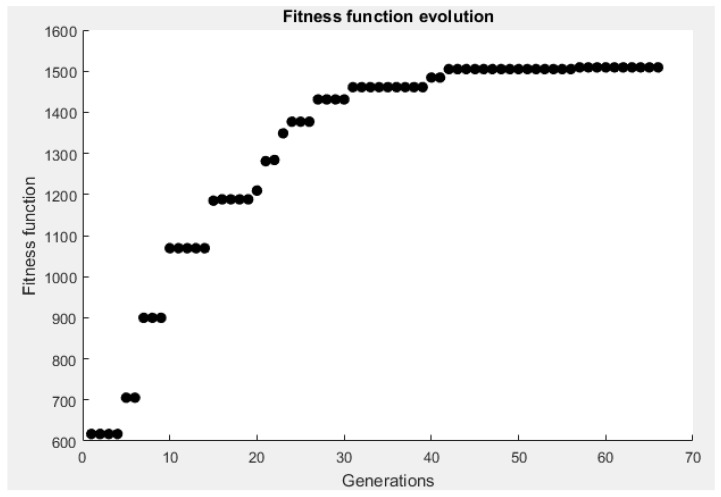
Evolution of the fitness function through several generations.

The process is performed iteratively until the *r* value is reduced. In the final step of the filter, more than 85% of the sample points are preserved and the representativeness is guaranteed, as is shown in Figure 5.

Previous studies have shown a clear relationship between the radius converging towards the correct solution in a four-sensor TDOA problem and a 3D-node distribution. Assuming this hypothesis is right, a 3D space will be associated with a certain node distribution that optimizes the convergence radius. 

This hypothesis was validated by means of optimization techniques, but presents two characteristics that dissuade resolution techniques based on exact algorithms—large solution space sizes (related to the required resolution level in sensor location) and an inability to apply recursive methodologies or separate the optimization into parts. Due to these circumstances, the optimization procedure is suitable to be performed by means of heuristic algorithms. Furthermore, Tekdas et al. [20] demonstrated that the node distribution problem is considered as NP-hard and must be solved with the usage of heuristic techniques.

Genetic algorithms represent a robust and flexible approach that allows the possibility of using non-derivable functions and an appealing trade-off between diversification and intensification in the solution-searching process of the problem. As an alternative to genetic algorithms, techniques such as randomized search, proposed by Bergstra and Bengio [21], are also suitable for approaching this problem. The positioning problem can be seen as an optimization problem where the size of the convergence spheres plays the role of loss function while the position of the beacons can be considered as hyperparameters for the underlying positioning algorithms. This paper focuses on reporting the results obtained by using genetic algorithms.

The starting point for an analysis is the definition of the 3D experimental volume of dimensions, 1000 × 400 × 100 m, described with a spatial discretization of 100 m in *x* coordinate, 50 m in the *y* coordinate and 10 m in the *z* coordinate. Each of the discretization points represents a real solution to the 3D TDOA system of study. Additionally, the height of the nodes has been limited to 15 m measured from the *z* = 0 plane, similar to the conditions found in a local, terrestrial positioning system.

The genetic algorithm developed for this study is based on binary codification techniques of the population, tournament-based selection, single-point crossover, 10% elitism and a mutation probability of 4%. The fitness function has been defined as the arithmetic mean of the distance between solutions for all points at the discretization, corrected according to parameter *r*.

The stop criterion of the algorithm has been defined as the instant when the maximum of the fitness function stops improving at the same time as the solution is reached for at least half of the individuals of the population. The resolution of the genetic algorithm is shown by means of the fitness function of the problem, in relation to the number of generations.

**Figure 6:**
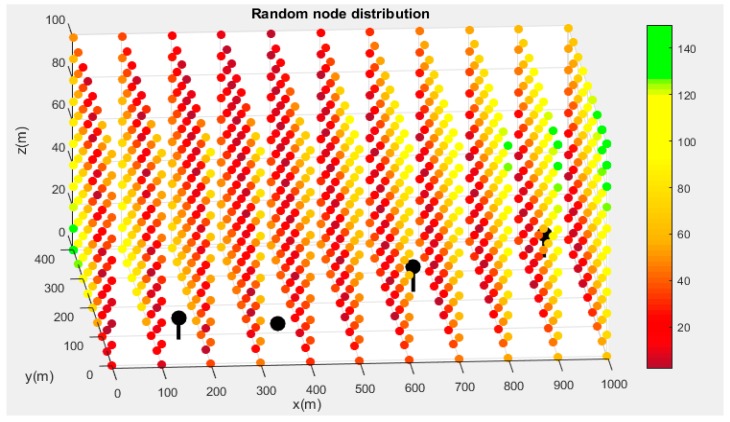
Evaluation of the convergence radius in the coverage area for a random distribution.

**Figure 7:**
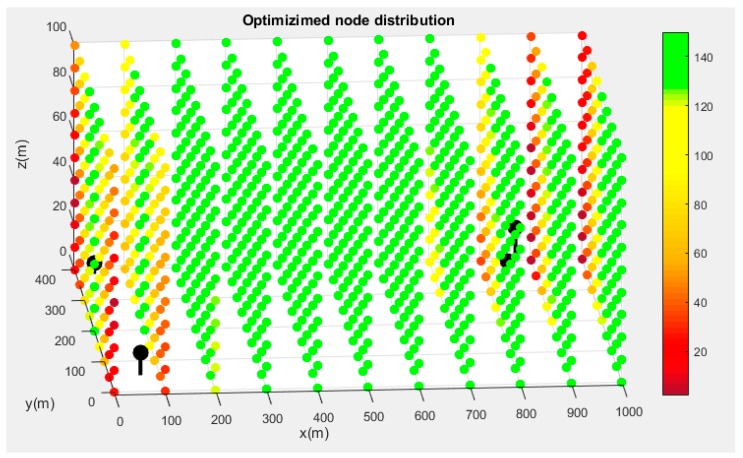
Evaluation of the convergence radius in the coverage area for the optimized distribution.

The final result of the process can be seen in Figure 6 and Figure 7. Figure 6 shows the evaluation of the convergence radius for the random distribution of points. The solution obtained after the maximization process is presented in Figure 7.

It is noteworthy to highlight the continuity presented by the convergence radius in all the domains and the negative influence they have on areas close to nodes. This is related to the geometry of the hyperboloids in these regions. In Table 2, a comparison between the distributions of nodes and the main statistical variables of the set of convergence radii is presented.

The results of the analysis lead to the conclusion that the initial hypothesis is correct, and hence a clear relationship exists between node distribution and the convergence radius of the four-node 3D-TDOA problem for the calculation of the position. Moreover, the whole procedure has been defined on the basis of genetic algorithms, making it possible to maximize the convergence radius in any environment, optimizing the product speed time refreshing rate. 

## 7. Discussion

A new methodology based on convergence properties of TDOA algorithms has been proposed in order to solve the four-sensor TDOA problem. This approach considers a procedure to maximize the capabilities of the algorithms in a confidence interval without considering the existence of errors due to signal transmission, signal processing or the synchronization of the system. For this reason, in future works it is necessary to consider optimization in a context where a Non-Line-of-Sight (NLOS) scenario is presented, clock synchronization is considered and other properties related to node distribution are also contemplated.

However, this paper presents a new perspective that concludes that algorithm properties are strongly related with node distribution and that the four-node TDOA problem can be solved under certain conditions with complete security for the first time in local positioning systems. With this optimization, convergence has also been maximized, which is one of the biggest problems of gradient descent algorithms in that they are deeply dependent on the initial iteration point [19]. In practice, this point is the last estimated position. The last position can be far away from the new target localization if the vehicle is moving at high speed, which can represent a convergence uncertainty. For this reason, a confidence region around the target localization has been defined to use the gradient descent algorithm under convergence conditions. The confidence region has been maximized through the radius of convergence and the calculation of the position has been guaranteed all over the domain in the optimized distribution, which does not happen in random distribution. This has important relevance in indoor positioning and precision landings in wide area multilateration, where sensor location must be considered. The reduction of one receiver guarantees system availability in cases of sensor failure, and reduces overall costs. 

## 8. Conclusions

In this paper, it has been shown that the TDOA problem can be solved with only four sensors within a confidence interval defined through the convergence radius. The great computational processing time needed to calculate this parameter has led to the search for another indicator—the distance between solutions, which permits a nearly complete explanation of the convergence radius.

This geometric factor must be filtered with the aim of allowing a statistical comparison between different node distributions. The high number of possible solutions has promoted the utilization of artificial intelligence through genetic algorithms, which have permitted the improvement of the convergence radius through optimized node distribution.

A comparison between a random and an optimized distribution shows the suitability of the methodology proposed to solve the TDOA problem with four sensors. By applying the sequential approximation algorithm between the two distributions, the confidence level is improved by over 400%. Furthermore, if a refresh rate of the positioning signal is fixed in one second, the algorithm can be used with four beacons in the optimized distribution 96.7% of the time with total security if the vehicle has a maximum speed of 25 m/s. In contrast, it can be used only in 31.2% of cases for random distribution.

The geometric statement of the problem of the intersection of hyperboloids has shown that an improvement in the space localization of the hyperboloids through node localization optimization allows the 4-sensor TDOA problem to transform into an analogous problem in which more receivers are used. This methodology ultimately provides great improvements to the positioning algorithm properties used throughout this article.

## Figures and Tables

**Table 1:** Correlation between radius of convergence and distance between solutions.

Parameter		Convergence Radius	Solutions Distance
Convergence radius	Pearson Correlation Coefficient (PCC)	1	0.999
	S. (bilateral)	-	0.000
	Samples	33,306	33,306
Solutions distance	Pearson Correlation Coefficient (PCC)	0.999	1
	S. (bilateral)	0.000	-
	Samples	33,306	33,306

**Table 2:** Statistical parameters of the optimized and random distribution.

Convergence Radius	Optimized Distribution	Random Distribution
Mean (m)	186.03	45.63
Min (m)	10	2
Max (m)	350	150
Std (m)	87.06	30.58
% Points convergence radius > 120	74.10%	1.56%
